# The Difference of Structural State and Deformation Behavior between Teenage and Mature Human Dentin

**DOI:** 10.1155/2016/6073051

**Published:** 2016-02-16

**Authors:** Peter Panfilov, Dmitry Zaytsev, Olga V. Antonova, Victoria Alpatova, Larissa P. Kiselnikova

**Affiliations:** ^1^Ural Federal University, Ekaterinburg 620083, Russia; ^2^Moscow State Medical Stomatological University, Moscow 127473, Russia

## Abstract

*Objective*. The cause of considerable elasticity and plasticity of human dentin is discussed in the relationship with its microstructure.* Methods*. Structural state of teenage and mature human dentin is examined by using XRD and TEM techniques, and their deformation behavior under compression is studied as well.* Result*. XRD study has shown that crystallographic type of calcium hydroxyapatite in human dentin (calcium hydrogen phosphate hydroxide Ca_9_HPO_4_(PO_4_)_5_OH; Space Group P63/m (176); *a* = 9,441 A; *c* = 6,881 A; *c*/*a* = 0,729; Crystallite (Scherrer) 200 A) is the same for these age groups. In both cases, dentin matrix is X-ray amorphous. According to TEM examination, there are amorphous and ultrafine grain phases in teenage and mature dentin. Mature dentin is stronger on about 20% than teenage dentin, while teenage dentin is more elastic on about 20% but is less plastic on about 15% than mature dentin.* Conclusion*. The amorphous phase is dominant in teenage dentin, whereas the ultrafine grain phase becomes dominant in mature dentin. Mechanical properties of human dentin under compression depend on its structural state, too.

## 1. Introduction

It is well-known that strength of teeth depends on the age of a human [1–4]. Many factors are responsible for this phenomenon, for example, metabolism, pathologies, and quality of food. Mechanical properties of a solid are sensitive to its structure and, therefore, an evolution of structure of the hard tissues of tooth during human life is also important factor, which should be taken into account. Dentin is the hard basis of a human tooth, which possesses complicated hierarchical structure [5, 6]. It consists of inorganic (approximately 50% by volume or 70% by weight) substances, organic (approximately 30% by volume or 20% by weight) substances, and water (approximately 20% by volume or 10% by weight) [7, 8]. The ratio between inorganic and organic components depends on human age: the portion of inorganic increases with age [9, 10]. Human dentin forms in about twelve years old and its structure continues to be the same on the macroscopic level during human life, whereas the width of root channels decreases [11, 12]. On the mesoscopic scale, the lumen of the dentin tubulars decreases and the width of peritubular dentin increases with age [1, 13]. Calcium hydroxyapatite in the ultrafine grain state (crystallite size 20–40 nm) is the mineral basis of dentin matrix on the microscopic level [14–17]. Despite considerable attention of researchers to structure and mechanical properties of the tooth hard tissues, the age dependence of the structural state of calcium hydroxyapatite in human dentin was not studied in detail [18–20]. It was shown that the portion of inorganics increases with age due to obliteration of dentin channels only, whereas intertubular dentin does not change, but sometimes the average size of crystallites of calcium hydroxyapatite decreases [2, 21, 22].

Human tooth is originated from the living cells as a soft tissue and, therefore, initially dentin should be in the amorphous/noncrystalline state [23, 24]. During human life, the structural state of calcium hydroxyapatite in dentin transforms from amorphous into the ultrafine grain state or, in other words, the amorphous matrix of dentin is crystallizing. According to the supposition about origination of human dentin, some of its properties should be similar to both soft tissue and crystalline solid. Indeed, mechanical behavior of human dentin is close to such filled polymers as a rubber and a low alloyed steel: under compression it exhibits the high elasticity (up to 40%) and considerable plasticity (up to 20%) at the high loads (up to 800 MPa) [25, 26]. However, the driving forces of this transformation, including the mechanism of nucleation of nanoscale grains, are still unclear. X-rays diffraction (XRD) and transmission electron microscopy (TEM) are the most appropriate methods for attestation of the structural state of a solid [27]. Such data allow doing the important step in the better understanding of the cause of considerable elasticity and plasticity of human dentin.

It is well-known that mechanical properties of some filled polymers are governed by elasticity of polymer matrix and hard particles of a filler, which cause growing of the strength but decreasing of elastic-plastic properties. The inclusion volume fraction and its size rule by the relationship between strength and deformability of the composite. Indeed, some mechanistic models of hard biological materials, including dentin, consider their microstructure as hard particles enveloped by a soft matrix [28, 29]. According to this model, calcium hydroxyapatite serves by filler in organic matrix and, hence, its structural state should rule by deformation behavior of human dentin. Since the moment when secondary dentin was formed, the inclusion volume fraction was changing insignificantly, whereas the grain size of calcium hydroxyapatite could vary. Teenage human dentin is the suitable model substance for the structural study of transition from amorphous to ultrafine grain state in the calcium hydroxyapatite, as it is the tissue approximating most of the initial state of secondary dentin. Information on the mechanical properties of teenage dentin allows understanding how such transition is reflected on deformation behavior of human dentin. Therefore, the aim of this work is XRD and TEM examination of structural state and study of deformation behavior under compression of teenage human dentin in comparison with mature dentin.

## 2. Materials and Methods

### 2.1. Samples Preparation

**Figure 1:**
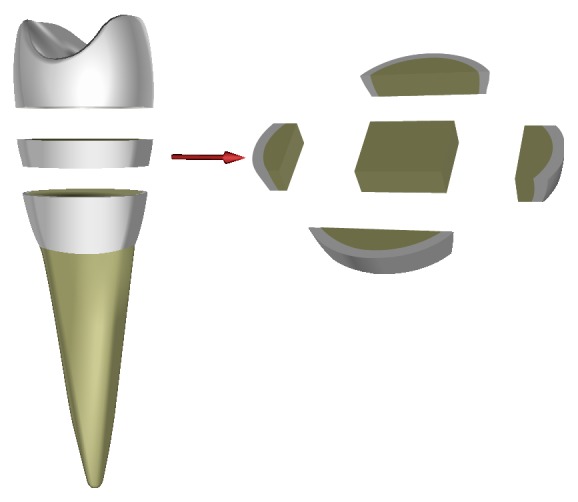
Scheme of cut-off tooth for preparation samples for structure study.

Thirty intact human premolars including upper and lower ones, which were extracted from the different subjects each according to the medical diagnosis and the Ethic Protocol of the Moscow State Medical Stomatological University, were used. Not any mechanical damage and pathologies in all teeth were observed. Two groups containing fifteen teeth each have been studied. The first group (teenage group) consists of the teeth of subjects of 12–16 years old, whose crowns and roots have been completely formed. The teeth of subjects of 30–40 years old were collected in the second group (mature group). Samples for examination of structure having the thickness of 0.5 mm were cut from the middle part of crown perpendicularly to the main tooth axis with the help of diamond saw under water irrigation according to the scheme in Figure 1. Enamel with mantle dentin was mechanically separated from the samples. This technique allowed preparing the samples with sufficient square from specified areas of a tooth.

### 2.2. XRD Analysis

After that every sample for the structural study was examined by the conventional X-ray diffraction (XRD) on the DRON4 X-rays diffractometer (Cu *k*_*α*_ irradiation). The beam width of 5 mm was pointed to the center of sample and almost covered it. Parameters of exposition were the following: accelerating voltage 30 KV; beam current 30 mA; the exposition 50 seconds per point; the step 0.1 degrees. Such regime excluded the burning of dentin under X-ray beam. The PDF-2*™* Database has been used for the analysis.

### 2.3. TEM Observation

Thin foils for TEM have been prepared by the following means. After X-rays examination, the samples were mechanically thinned down to 0.1 mm in thickness. Abrade papers and pastes having grits 1500 were applied for this procedure. The final thinning of samples was carried out by the chemical polishing in the jet of concentrated H_3_PO_4_ acid for 40 minutes. Thin foils possessed smooth surfaces without etching pits and the thickness, which was sufficient for TEM observation at electron beam of 200 KV. Further thin foils were washed in the water jet for 30 minutes and dried on air. At the end, the foils were fixed on the copper grid by the conducting glue. This procedure prevented folding of the samples under electron beam. TEM study of dentin has been carried out on JEM-2010 (200 KV) with site entry of samples meant for materials science researches. It should be noted that described technique allowed observing microstructure of calcium hydroxyapatite but not organic compounds in dentin. No ion milling was applied for the thin foil preparation, insomuch as it induced a lot of irradiation damage in dentin.

### 2.4. Mechanical Testing

Ten samples for uniaxial compression test having the size of 2 × 2 × 0.65 mm^3^ were prepared from the crown parts of teeth of each group according to procedure previously described [26]. The diamond saw under water irrigation was used for cutting of the teeth. Back surfaces of the samples were mechanically abraded by means of the abrasive papers for removing of the damaged layer that could appear under cutting off procedure. Room temperature compression tests were carried out on Shimadzu AGX-50 kN testing machine at the traverse rate 0.1 mm/min. The Trapezium*™* software was used for the processing of experimental data.

## 3. Results

### 3.1. XRD Study

**Figure 2:**
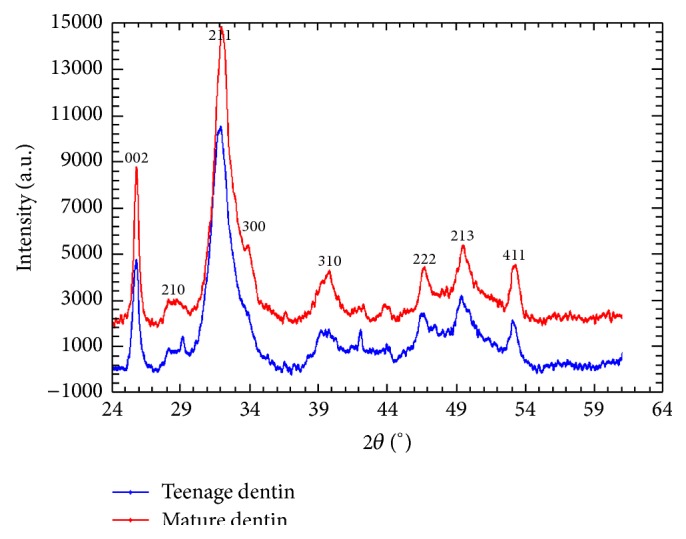
XRD specter of teenage and mature human dentin. In both cases human dentin is in X-ray amorphous state.

The typical XRD specters taken from the samples belonging to both teenage and mature group were shown in Figure 2. No significant differences between them were observed. All peaks in the specters were considerably widened and, hence, the structural state of dentin in these groups may be characterized as X-ray amorphous [16, 30]. In spite of this feature of the peaks, the analysis of X-ray specters could be carried out. It has shown that the inorganic basis of teenage and mature dentin was calcium hydroxyapatite in the hexagonal form (Ca_9_HPO_4_(PO_4_)_5_OH; Space Group P63/m (176); *a* = 9,441 A; *c* = 6,881 A; *c*/*a* = 0,729; Crystallite (Scherrer) 200 A) [31, 32].

### 3.2. TEM Study

**Figure 3:**
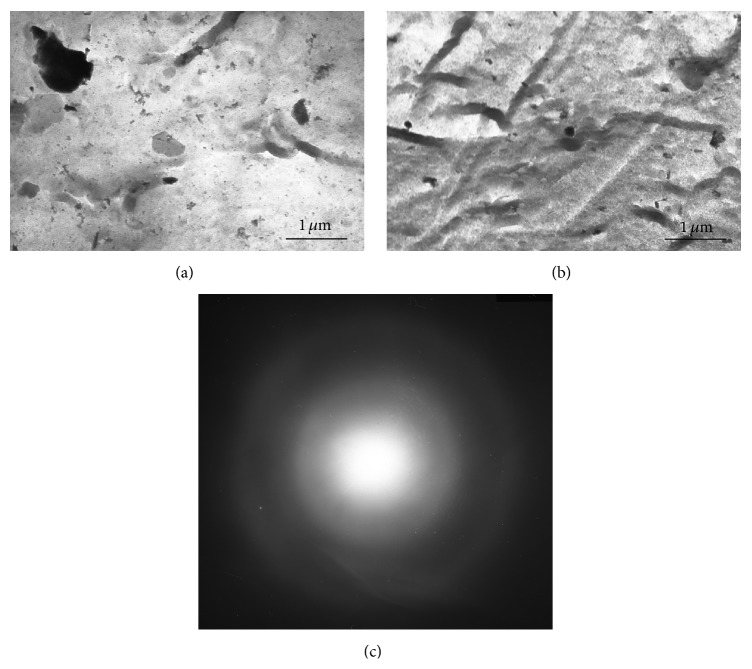
TEM microstructure of teenage dentin, bright field images; (a) image contained hexagonal crystals; (b) image contained traces of collagen fibers; (c) electron pattern. It is in the amorphous state.

Clear bright field TEM images at low magnifications (≤×10 000) were obtained in teenage dentin foils (Figures 3(a) and 3(b)). According to the diffuse halo on the electron diffraction patterns (Figure 3(c)), calcium hydroxyapatite of teenage dentin was in the amorphous state. No spots on the electron diffraction were observed. Therefore, clear focused images at the higher magnifications could not be taken in these foils [33, 34]. TEM images of teenage dentin represented dark and gray plane inclusions on light tweed-contrasted matrix (Figure 3(a)). The shape of some inclusions was close to a hexagon. The size of small hexagonal inclusions was about 50 nm, whereas the big ones could reach up to 500 nm. Another morphological feature of microstructure of these foils was randomly oriented dark ribbons having the width about 100 nm (Figure 3(b)).

**Figure 4:**
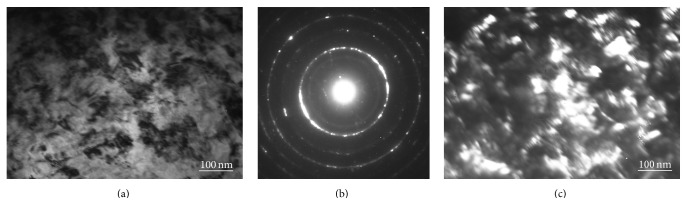
TEM microstructure of mature age dentin: (a) bright field, (b) electron pattern, and (c) dark field. It is in the ultrafine grain state.

In contrast with teenage group, no clear focused bright field images were obtained from thin foils of the mature group at low and middle range of magnifications (≤×30 000), in spite of the thin foils being transparent for 200 KV electron beam. Satisfactory focused bright field images were taken at high magnification only (≥×50 000) (Figure 4(a)). Dark inclusions having complicated shape and size which varied from 10 nm to 100 nm were visible on the light gray field. The ratio between dark and light gray areas on the images was about 1 : 1. No traces of collagen fibers were observed here. Both diffuse halo and diffraction spots, which had a tendency to join in the rings, were clearly visible on the electron pattern (Figure 4(b)). Estimation of the dark field images (Figure 4(c)) has shown that some inclusions in mature dentin had size of 10–100 nm.

### 3.3. Compression Test

**Figure 5:**
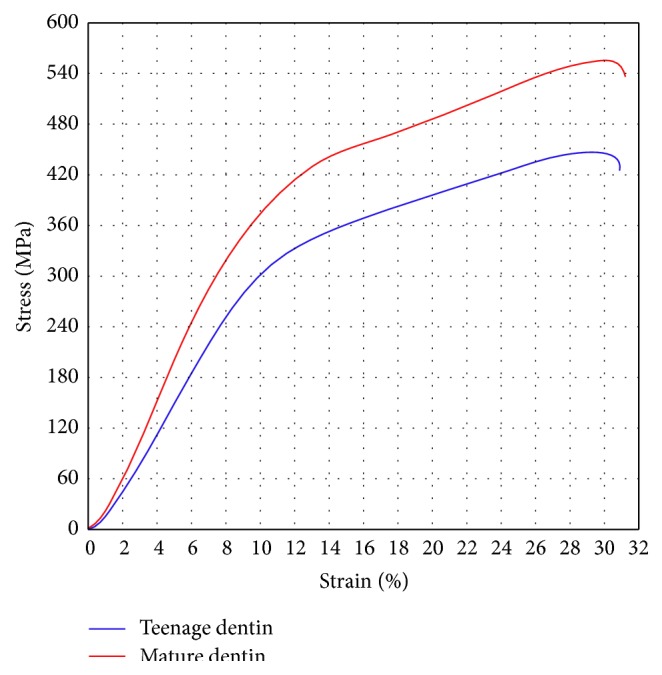
Typical stress-strain curves under compression: red curve, teenage dentin; blue curve, mature dentin. Compression strength of teenage dentin is less on about 20% than mature dentin, while their total deformation is the same.

The typical compression curves of teenage and mature dentin, which were close to the average ones, were presented in Figure 5. No qualitative difference in deformation behavior between them was observed. The samples never failed under the testing in spite of cracks appearing in them. There were two almost linear regions that differed by the slope in curve. Young's modulus has been calculated from the slope of the first linear region. Compression strength and total deformation have been obtained from the point of the maximal stress in the curve. Plastic deformation of the samples has been calculated as the difference between its height in the initial state and after compression, while their elastic deformation has been determined as the difference between total and plastic deformation. The findings were collected in Table 1. It was clearly visible that Young's modulus and compression strength of teenage dentin were less on about 20% than mature dentin. Although the total deformation was the same for both cases, the contributions of elasticity and plasticity were different. Teenage dentin was more elastic tissue on about 20%, while mature dentin was more plastic tissue on about 15%. Analysis of the trend of deformation curves has shown that the first linear region of curve and the transition region between the first and the second linear regions of curve were elastic for teenage and mature dentin. The second linear region of curve was plastic region for mature dentin and elastic-plastic region for teenage dentin. However, in both cases human dentin was highly elastic, plastic, and strong substance.

## 4. Discussion

XRD findings give the basis for conclusion that both teenage and mature dentin consist of the sole type of calcium hydroxyapatite, in which structural state is X-ray amorphous. As a rule, the similar structural state is observed in materials consisting of the number of small areas, whose structural states vary from amorphous to ultrafine grain state. Hence, the difference between inorganic bases of teenage and mature dentin can be only in proportion between amorphous and ultrafine grain phases of calcium hydroxyapatite, which, however, cannot be detected by conventional XRD technique giving average information on microstructure of a sample.

TEM is also diffraction method, but it allows estimating the microstructure of small local areas in thin foils. It has shown that teenage dentin is mainly in amorphous state. It may be supposed that hexagonally grained inclusions, observed in the amorphous matrix, are the thin crystallites of hexagonal calcium hydroxyapatite on the basal plane (Figure 3(a)). Hence, calcium hydroxyapatite in teenage dentin is the mixture of amorphous and crystalline phases. However, the quantity of these crystallites (crystalline phase) is small in comparison with amorphous phase (tweed-contrast matrix) and its contribution to the diffraction pattern of teenage dentin is insufficient and does not lead to appearance of diffraction spots. The small quantity of the crystalline phase does not allow forming the diffraction contrast images from thin foil and, as a result, the high magnification images of young dentin cannot be taken [35]. The dark ribbons in the tweed-contrast matrix may be determined as the traces of collagen fibers in human dentin, because the fibers have similar size and orientation (Figure 3(b)) [14, 36, 37]. TEM study of mature dentin shows that ultrafine grains are its dominant structural state, whereas the amorphous phase is almost absent in thin foils. It should be noted that ultrafine grain state of the crystalline phase of calcium hydroxyapatite does not allow forming the clear diffraction contrast image at low magnifications [33]. Collagen fibers are never observed in the thin foils of mature dentin, inasmuch as the TEM images have been formed in the diffraction contrast, while the TEM images of teenage dentin have been formed in the diffusion contrast.

Therefore, it may be concluded that initially calcium hydroxyapatite of human dentin is amorphous (at teenage), but further it begins crystallizing until mature age when dentin is fully in ultrafine grain state. Tiny crystallites of calcium hydroxyapatite nucleate and grow in the amorphous matrix of teenage dentin. However, it is special kind of crystallization, insomuch as the average size of crystallite is limited by 100 nm as it was mentioned in the literature [2, 21].

The experimental data on the structural state of teenage and mature dentin may be helpful for the better understanding of the deformation behavior of human dentin. Synthesized or natural calcium hydroxyapatite behaves like hard rock material: it is practically undeformed and brittle [38–41]. In contrast with this, human dentin is biomineral, in which elastic and plastic properties are similar to those of rubber, while its strength is close to synthesized calcium hydroxyapatite [42–44]. Considerable part of organic compounds in dentin may be considered as the cause of such unusual deformation behavior. For example, elastic-plastic collagen fibers partially provide elasticity and plasticity of dentin [45, 46]. On the contrary, the tiny particles of calcium hydroxyapatite inside organic matrix serve as filler and keep the strength to dentin. Compression tests have shown that the structural state of calcium hydroxyapatite influences the mechanical properties of dentin. Mature dentin, where calcium hydroxyapatite was in the ultrafine grained size, is stronger on about 20% than amorphous teenage dentin, but their ability of elastic-plastic deformation continues to be the same. The elasticity of teenage dentin is higher on about 20% and its plasticity is lower on about 15% in comparison with mature dentin. Consequently, these features are connected with the size of the particles of filler, the grain size of calcium hydroxyapatite, as it takes place in filled polymers [47].

## 5. Conclusions

Microstructure of human dentin is the mixture of amorphous and ultrafine grain phases whose ratio is cardinally changing from teenage to mature dentin. Amorphous phase is dominant in teenage dentin and, on the contrary, crystalline phase becomes dominant in mature dentin. The mechanical properties of human dentin depend on its structural state, too. Young's modulus and compression strength of teenage dentin are less on about 20% than mature dentin, while their total deformation is the same. At that teenage dentin is the more elastic tissue on about 20%, but mature dentin is the more plastic tissue on about 15%.

## Figures and Tables

**Table 1:** The mechanical properties with standard deviation of teen and mature age dentin under compression.

Type of dentin	Young's modulus, GPa	Compression strength, MPa	Elastic deformation, %	Plastic deformation, %	Total deformation, %
Teenage	3.43 ± 0.13	445.2 ± 30.9	16.8 ± 1.2	14.5 ± 1.6	30.3 ± 1.5
Mature	4.44 ± 0.46	549.5 ± 26.9	13.4 ± 0.9	17.0 ± 4.0	30.4 ± 3.3
